# Clinicopathological significance of fascin and CD44v6 expression in endometrioid carcinoma

**DOI:** 10.1186/1746-1596-7-80

**Published:** 2012-07-11

**Authors:** Banu Dogan Gun, Burak Bahadir, Sibel Bektas, Figen Barut, Gamze Yurdakan, Nilufer Onak Kandemir, Sukru Oguz Ozdamar

**Affiliations:** 1 Department of Pathology, Faculty of Medicine, Bulent Ecevit University, 67100 Kozlu, Zonguldak, Turkey

**Keywords:** Fascin, CD44v6, Endometrioid carcinoma, Immunohistochemistry

## Abstract

**Background:**

Fascin and CD44v6 may have significant roles as biomarkers in tumour progression and metastasis. In endometrioid carcinomas, the fascin expression profile is less defined, and the significance of CD44v6 is uncertain. We aimed to investigate the expressions of both fascin and CD44v6 in endometrioid carcinomas and to evaluate their inter-relation with clinicopathological parameters.

**Methods:**

Fascin and CD44v6 expressions were evaluated, individually and in combination, in a series of 47 endometrioid carcinomas and 10 proliferative endometrium samples. The staining extent and intensity of both markers in tumour cells were scored semiquantitatively. The relationship between immunoexpressions and clinicopathological variables was assessed.

**Results:**

The expression rates of fascin and CD44v6 in endometrioid carcinoma were 72.34% and 46.80%, respectively. Although these expression rates were higher than those in proliferative endometrial samples, fascin expression showed a statistically significant difference from the normal group (*p* = 0.02), but CD44v6 did not differ (*p* = 0.54). Fascin expression was significantly correlated with tumour grade (*p* = 0.003) and neural invasion (*p* = 0.036) in a univariate analysis. In contrast, no significant correlation was found between CD44v6 and any of the clinicopathological parameters.

**Conclusions:**

Our findings suggest that fascin might be an independent prognostic indicator in the different steps of extracellular matrix invasion. On the other hand, CD44v6 was not a predictive factor in endometrioid cancer.

**Virtual Slides:**

The virtual slide(s) for this article can be found here: http://www.diagnosticpathology.diagnomx.eu/vs/8511594927206899.

## Background

Endometrial carcinoma is the most common invasive neoplasm in the female genital tract. Based on clinicopathological and molecular genetic features, it can be divided into two major groups referred to as type I and type II. The endometrioid subtype, which is the prototype of type I carcinoma, is associated with unopposed estrogenic stimulation, as well as endometrial hyperplasia. Prognosis is dependent on some well-accepted clinical and pathological parameters, including the histological type and grade of the tumour, the depth and pattern of myometrial invasion, the degree of disease extension beyond the uterine corpus, adnexal involvement, and pelvic and para-aortic lymph node metastasis [1]. Although several common molecular alterations have been identified in the pathogenesis of endometrial cancer [2-4], additional molecular factors need to be defined to predict the specific behaviour of the tumour.

The interaction between epithelial tumour cells and their stroma is very crucial in tumour progression and metastatic cascade. To escape from the primary tumour and invade adjacent tissues, cancer cells must interact with the extracellular matrix at several stages [5]. Invasive cancer cells are believed to breach the basal membrane using specialised protrusions called invadopodia [6-8]. The forces that drive tumour cell migration and invasion are provided by the actin cytoskeleton underlying the membrane protrusions [9,10]. Fascin is a 55 kDa actin-bundling protein and is an important regulatory element in the maintenance and stability of parallel bundles of filamentous actin in a variety of cellular contexts. The ability of fascin to bind and bundle actin plays a central role in the regulation of cell adhesion, migration, and invasion [11].

CD44 is a transmembrane receptor protein that belongs to the family of adhesion molecules and is expressed on the surface of diverse cell types [12]. This glycoprotein has a critical role in extracellular matrix adhesion and is implicated in a series of cellular events, such as lymphocyte homing, leukocyte activation, lymphopoiesis, embryogenesis, and wound healing [13]. CD44 is encoded by a single gene containing 20 exons, 10 of which (v1–v10) are variant exons [14]. The overexpression of the variant form, CD44v6, which contains sequences encoded by exon 6, has been proposed as a potential prognostic marker in many epithelial and nonepithelial malignancies in both early and metastatic phases of carcinogenesis [13].

Fascin expression has been evaluated in several human neoplasia, and recently in epithelial tumours [15-30], but to our knowledge only two studies have been conducted on the significance of fascin expression in endometrioid carcinomas [31,32]. With regard to CD44 and its variants, several studies have investigated its expressions in endometrial pathologies, including adenocarcinomas, yielding different results [31-41]. Our purpose was to evaluate the possible roles of these two molecular markers, fascin and CD44, in the invasive and metastatic behavior of endometrioid carcinoma and to analyse their association with clinicopathological features.

## Methods

### Patients

We studied 47 well-documented cases of endometrioid adenocarcinoma for which archival material of surgical specimens from primary tumour resections were available between 2006 and 2011 at the Pathology Department of Bulent Ecevit University Hospital. Tumours were staged according to the 2010 FIGO (The International Federation of Gynecology and Obstetrics) staging system. The hematoxylin and eosin stained sections were reviewed to determine the FIGO grade, the depth of myometrial invasion (if present), and the presence or absence of lymphovascular invasion and neural invasion. The macroscopic sizes and the FIGO stage of all tumours were also noted. Proliferative phase endometrium tissue samples were obtained from 10 cases.

### Immunohistochemical staining

The sections (4–5 μm) obtained from representative tissue sample blocks were deparaffinised with xylene, rehydrated in graded alcohols, and placed in 0.5% hydrogen peroxide in methanol for 10 min to block endogeneous peroxidase activity. Antigen retrieval was carried out by incubation in 0.01 M citrate buffer (ph 6.0) for 5 min in a pressure cooker. The sections were exposed to the primary antibody for 60 min at room temperature. The standard streptavidin-biotin-peroxidase complex method was used for fascin (IM20, Novocastra, Newcastle, UK, 1:400) and CD44v6 (VVF-7, Novocastra, Newcastle, UK, 1:50) by employing diaminobenzidine (DAB) as the chromogen. Human tonsil was used as a positive control, while negative controls were obtained by omitting the primary antibody.

### Evaluation of immunohistochemical staining

We calculated the “immunohistochemical score” (IHS) of fascin and CD44v6 for each case. The scoring system used for both fascin and CD44v6 was similar to previously published methods [20,40]. The extent of positively stained epithelial cells was estimated and classified on a four-point scale as follows: no staining = 0%, 1 = 1%–10%, 2 = 11%–25%, 3 = 26%–50%, and 4 = 51%–100%. The intensity of the immunoexpression was categorised into three groups: weak (+1), moderate (+2), and strong (+3). A final IHS score was obtained by multiplying the score for extent and the score for intensity. Therefore, the combined immunoreactivity score ranged from 0 to 12. According to this, cases were categorised into three groups: 0 (absent), IHS ≤ 10, and IHS ≥ 11.

### Statistical analysis

Statistical analysis was conducted with SPSS 18.0 software (SPSS, Inc., Chicago, IL). Continuous variables were expressed as mean ± standard deviation, and categorical variables were expressed as frequency and percentage. Pearson’s chi-square test was used to determine the difference between groups. A *p* value of less than 0.05 was considered statistically significant for all tests.

## Results

### Immunohistochemical expression of fascin

The median age of the patients was 58.60 (SD ± 11.16) years, with a range of 34–79 years. The macroscopic tumour sizes varied from 1 to 9, with a mean size of 4.41 cm (SD ± 11.16). According to the FIGO staging system, 19 tumours were in stage 1A, 11 in stage 1B, 11 in stage 2, 3 in stage 3, and 4 in stage 4. Histopathologically, there were 16 grade 1, 16 grade 2, and 15 grade 3 tumours.

**Figure 1:**
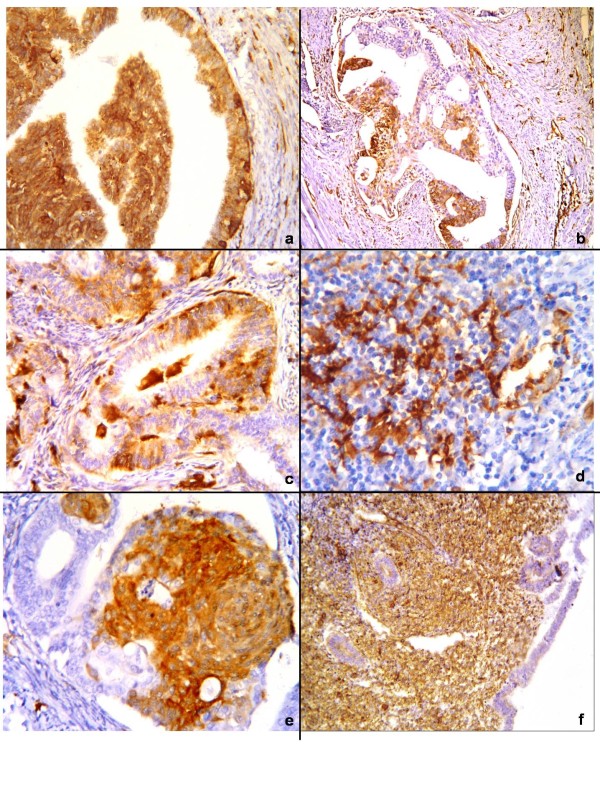
**Immunohistochemical staining for fascin.** (**a**) Strong fascin expression in tumour glands. (**b**) Heterogeneous expression was seen in the same gland. (**c**) Areas where immunoreactivity was seen at the peripheral portion of the glands. (**d**) The expression in an MELF-type area in a grade 2 tumour. (**e**) The expression in the areas of squamous differentation. (**f**) Diffuse staining of stroma in proliferative endometrium. B-SA peroxidase, DAB: [(**a**) and (**c**)] × 200, [(**b**), (**e**), and (**f**)] × 100, and (**d**) × 400.

Fascin was detected in 72.34% (34 out of 47) of the cases in the tumour cell cytoplasm. Also, microvessel endothelium was stained with fascin in all carcinoma cases. In the tumour stroma only a few inflammatory cells including histiocytes showed weak fascin immunoreactivity. Epithelial staining was heterogeneous; while a score ≤10 was detected in 56.25% (27) of the cases, a score ≥11 was only seen in 16.66% (7) of the cases. The positively stained epithelial cells with fascin revealed various staining patterns, such as diffuse expression in a gland, patchy staining in a gland, or aggregation at the peripheral portion of a gland [Figures (a)–1(c)]. There was no obvious micro-anatomical distribution of fascin expression in terms of superficial portion and conventional invasive areas. In our series, only one case (grade 2) exhibited a microcystic, elongated, and fragmented (MELF)-type invasion pattern and fascin was expressed strongly in the neoplastic epithelium within these areas [Figure 1(d)]. The foci of squamous/morular differentiation were all strongly stained with fascin [Figure 1(e)].

In the proliferative endometrium, the stroma stained diffusely and homogeneously in all samples. However, glandular epithelium stained weakly in three (30%) of the samples [Figure 1(f)]. A statistically significant difference with fascin expression was found between endometrioid carcinoma cases and proliferative endometrial samples (*p* = 0.023) (Table 1).

**Table 1:** Comparison of cases with endometrioid carcinoma and proliferative endometrium

	**IHS score**	**Endometrioid**	**Proliferative**	***P*value**
		**carcinoma**	**endometrium**	
		**(n = 47)**	**(n = 10)**	
**Fascin**	0	13 (27.7%)	7 (70.0%)	*p* = 0.023
	≤10	27 (57.4%)	3 (30.0%)	
	≥11	7 (14.2%)	0 (0%)	
**CD44v6**	0	25 (53.2%)	6 (60.0%)	*p* = 0.542
	≤10	19 (40.4%)	4 (40%)	
	≥11	3 (6.4%)	0 (0%)	

IHS: immunohistochemical score.

Fascin expression was found to be significantly correlated with tumour grade (*p* = 0.003) and with neural invasion (*p* = 0.036) (Table 2). This finding was only seen on a univariate analysis. As these variables were highly correlated (multicollinearity), binary logistic regression analysis did not fit the model and couldn’t be done. However, no correlation was detected between fascin expression and tumour size, degree of myometrial invasion, lymphovascular invasion, and tumour stage (*p* > 0.05).

**Table 2:** Correlation of clinicopathological parameters with fascin and CD44v6 expressions in 47 endometrioid carcinoma

**Variables**	**Fascin**				**CD44v6**			
	**Absent n = 13 (%)**	**IHS ≤ 10 n = 27 (%)**	**IHS ≥ 11 n = 7 (%)**	***P value***	**Absent n = 25 (%)**	**IHS ≤ 10 n = 19 (%)**	**IHS ≥ 11 n = 3 (%)**	***P value***
**Mass size**								
≤4	7 (53.8%)	13 (48.1%)	3 (42.9%)	0.889	12 (48.0%)	9 (47.4%)	2 (66.7%)	0.814
≥5	6 (46.2%)	14 (51.9%)	4 (57.1%)		13 (52.0%)	10 (52.6%)	1 (33.3%)	
**Grade**								
G1	9 (69.2%)	7.(25.9%)	0 (0%)	0.003	11 (44.0%)	5 (26.3%)	0 (0%)	0.322
G2	3 (23.1%)	11 (40.7%)	2.(28.6%)		7 (28.0%)	8 (42.1%)	1.(33.3%)	
G3	1 (7.7%)	9 (33.3%)	5 (71.4%)		7 (28.0%)	6 (31.6%)	2 (27.7%)	
**Depth of MI**								
Absent	2 (15.4%)	0 (0%)	0 (0%)	0.244	2 (8.0%)	0 (0%)	0 (0%)	0.527
Superficial	5 (38.5%)	13 (48.1%)	3 (42.9%)		10 (40.0%)	9 (47.4%)	2 (66.7%)	
Deep	6 (46.2%)	14 (51.9%)	4 (57.1%)		13 (52.0%)	10 (52.6%)	1 (33.3%)	
**LVI**								
Absent	9 (69.2%)	17 (63.0%)	2 (28.6%)	0.182	13 (52.0%)	12 (63.2%)	3 (100.0%)	0.150
Present	4 (30.8%)	10 (37.0%)	5 (71.4%)		12 (48.0%)	7 (36.8%)	0 (0%)	
**NI**								
Absent	11(84.6%)	26 (96.3%)	4 (57.1%)	0.036	22 (88.0%)	16 (84.2%)	3 (100.0%)	0.613
Present	2 (15.4%)	1 (3.7%)	3 (42.9%)		3 (12.0%)	3 (15.8%)	0 (0%)	
**Stage**								
1	8 (61.5%)	18 (66.7%)	3 (42.9%)	0.597	17 (68.0%)	10 (52.6%)	2 (66.7%)	0.084
2	3 (23.1%)	6 (22.2%)	2 (28.6%)		3 (12.0%)	8(42.1%)	0 (0%)	
3	1 (7.7%)	2 (7.4%)	0 (0%)		2 (8.0%)	0 (0%)	1 (33.3%)	
4	1 (7.7%)	1 (3.7%)	2 (28.6%)		3 (12.0%)	1 (5.3%)	0 (0%)	

IHS: immunohistochemical score, MI: myometrial invasion, LVI: lymphovascular invasion, NI: neural invasion.

### Immunohistochemical expression of CD44v6

**Figure 2:**
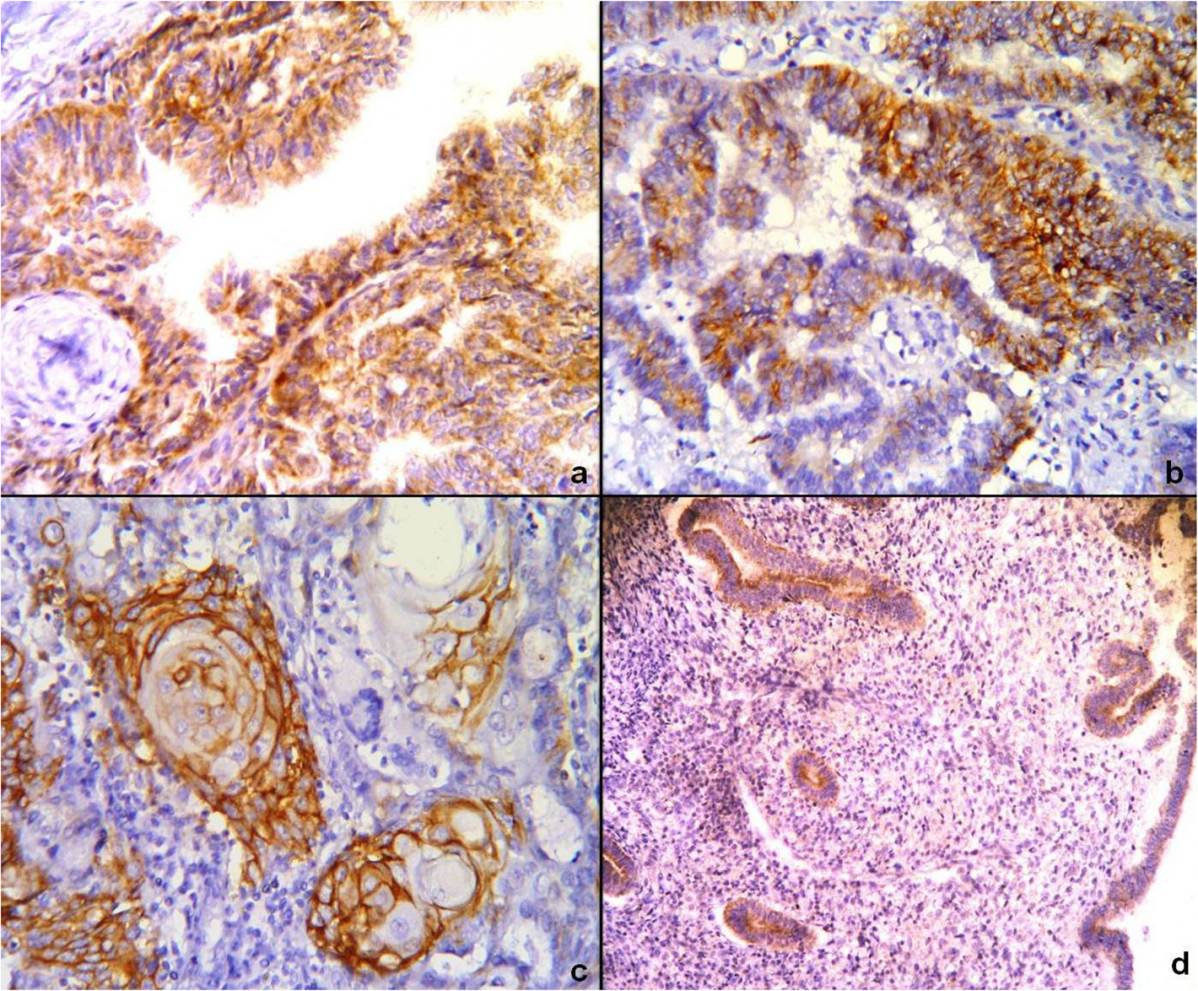
**Immunohistochemical staining for CD44v6.** (**a**) Cytoplasmic expression in tumour glands. (**b**) Focal membranous staining in the tumour. (**c**) Squamous differentation areas showing strong expression. (**d**) Weak expression in the glands of proliferative endometrium. B-SA peroxidase, DAB: [(**a**)–(**c**)] × 200 and (**d**) × 100.

The neoplastic glands in 46.80% (22 out of 47) of the cases showed cytoplasmic and membranous CD44v6 reactivity. The CD44v6 staining pattern was observed as cytoplasmic and/or membranous in tumour cells [Figure 2(a) and 2(b)], but cytoplasmic expression was the predominant pattern. In squamous/morular differentiation areas, however, membranous expression was strong and widespread [Fig. 2(c)]. The staining was not diffuse, and the total IHS score was calculated as ≥11 in only three tumours. No CD44v6 expression was seen within the MELF-type neoplastic epithelium, and no special micro-anatomical distribution was observed. Also stromal tumour cells were not stained with CD44v6. In four (40%) of the proliferative endometrial samples, CD44v6 expression was weak only in the glandular epithelium [Fig. 2(d)]. Statistically, no difference was found between endometrioid carcinoma cases and proliferative endometrial samples (*p* = 0.542).

Additionally, no significant correlation was detected between the expression of CD44v6 and any clinicopathological features of endometrioid carcinoma cases, such as tumour size, tumour grade, depth of myometrial invasion, lymphovascular invasion, neural invasion, and stage (*p* > 0.05).

## Discussion

Fascin and CD44 are proteins involved in different steps in the extracellular matrix invasion. The interaction between epithelial and stromal cells is important in tumour progression and metastasis; cancer cells use invasive fingerlike protrusions called invadopodia to invade the basal membrane and to degrade the extracellular matrix [6]. These protrusions carry actin bundles under the membrane [6,9,10]. At that point, fascin, the so-called actin-bundling protein, becomes a part of this process and is essential for the stability of actin microfilaments. It also facilitates the invasion [9].

Experimental studies demonstrated that CD44 was strongly associated with the actin cytoskeleton in an indirect position, and this interaction is mediated by ERM (ezrin-radixin-moesin) proteins [14,42,43]. ERM adapts CD44 to the actin-based cytoskeleton and organises the membrane and cytoskeleton interaction. However, little knowledge is known about the relationship between fascin and CD44 in solid tumours. In the present study, we examined fascin along with CD44v6 in 47 endometrioid carcinoma and 10 proliferative endometrium samples. We demonstrated that the expression of these two molecular proteins did not correlate with each other.

Fascin has recently received considerable attention as a new prognostic marker in several solid neoplasms. In the present study, we detected fascin in 72.34% of endometrioid carcinoma, and the expression was significantly different from the normal group. Among the studies about fascin in endometrial carcinomas, Kabukcuoglu et al. revealed positive staining in 74% of the carcinoma specimens and in 39% of the non-neoplastic endometrial samples [31]. They also indicated that higher-grade endometrial carcinoma cases revealed a significant increase in the total epithelial fascin expression. These results are considerably compatible with our findings that fascin was significantly correlated with histological grade. Stewart et al. also demonstrated strong fascin immunoreactivity in the neoplastic epithelium of MELF-type invasion areas in contrast to negative or weakly stained conventional tumour glands in low-grade uterine endometrioid adenocarcinoma. In addition, prominent staining within the peripheral epithelial cell and in the foci of squamous/morular-type differentiation was observed [32].

Of the 47 cases, only one (a grade 2 tumour) showed an MELF-type invasion pattern and revealed strong fascin expression. In a unique study determining the exact nature of the MELF pattern of invasion, the immunprofile in MELF areas was found to be consistent with the features of epithelial-mesenchymal transition [44].

Although areas of squamous or morular differentiation are frequently observed in endometrioid carcinomas, their pathogenesis remain unclear. In studies that investigated the precise nature of squamous differentiation in endometrial carcinoma, these areas were found to express beta catenin [45-47]. In our series, areas with squamous and morular differentation expressed both fascin and CD44v6 strongly. CD44v6 expression can be detected in a subset of squamous cell carcinoma in different locations and in the foci of squamous differentation [41,48,49]. This finding was attributed to the fact that the cells were packed tightly in these foci, as seen in squamous cell carcinoma of any organ or in solid nests within an otherwise adenocarcinoma [49]. Meanwhile the presence of fascin expression in squamous and morular differentiation was explained by the activation of the Wnt signalling pathway and the upregulation of fascin via nuclear translocation of beta catenin [32].

The immunoexpression of fascin and CD44v6 was predominantly heterogeneous, while diffuse and strong expressions were seen in 6.4% and 14.2% of cases, respectively. Fascin immunostaining was sometimes restricted to the periphery of the neoplastic glands. However, no special staining distribution was seen between the glands in the superficial portion of the tumour and in the deep invasive conventional areas. Being a dynamic process, carcinogenesis might be responsible for the heterogeneity of protein expressions.

CD44v6 has been implicated in the malignant transformation and metastatic potential of several tumours and in endometrial carcinomas. Among the previous analyses of CD44 in endometrial adenocarcinoma, some investigators found no relation between CD44v6 and clinicopathological parameters and thought that CD44v6 was not an adverse predictive factor [33-35]. However, the others indicated that CD44v6 was inversely correlated with adverse prognostic factors [37,38]. In this study, CD44v6 was demonstrated in 46.80% of carcinoma specimens and in 40% of the proliferative phase. Meanwhile, no correlation was found between any of the clinicopathological features. The expression of CD44v6 may be seen as a result of disregulation and is not primarly implicated in the progression of endometrial cancer.

## Conclusions

Our findings suggest that fascin may become a novel marker in the prognosis of endometrial cancer. However, further detailed studies in a larger series that should include prospective clinicopathological analyses and menstrual cycle phases are neccessary to determine the significance of these molecules in physiological conditions and in endometrial tumors as a prognostic indicator or a molecular target for treatment.

## Abbreviations

IHS, Immunohistochemical score; MELF, Microcystic, elongated, and fragmented; FIGO, The International Federation of Gynecology and Obstetrics.

## Competing interests

The authors declare that they have no competing interests.

## Authors’ contributions

BDG conducted the study design, performed microscopic evaluation, and drafted the manuscript. BB participated in the design of the study and helped to draft the manuscript. SB participated in the design of the study and performed microscopic evaluation. FB, GY, and NOK participated in the microscopic and immunohistochemical evaluation. SOO participated in the study design and coordination. All authors read and approved the final manuscript.
